# *Passiflora mollissima* Seed Extract Induced Antiproliferative and Cytotoxic Effects on CAL 27 Spheroids

**DOI:** 10.1155/2022/4602413

**Published:** 2022-05-31

**Authors:** Angela Fonseca-Benitez, Sandra Johanna Morantes Medina, Diego Ballesteros-Vivas, Fabian Parada-Alfonso, Sandra J Perdomo

**Affiliations:** ^1^Cell and Molecular Immunology Group, Universidad El Bosque INMUBO, Bogotá D.C., Colombia; ^2^School of Dentistry, Universidad El Bosque, Bogotá D.C., Colombia; ^3^Grupo de Investigación en Química Aplicada-INQA, Programa Química Farmacéutica Universidad El Bosque, Bogotá D.C., Colombia; ^4^Unit of Basic Oral Investigation (UIBO), School of Dentistry, Universidad El Bosque, Bogotá D.C., Colombia; ^5^Departamento de Nutrición y Bioquímica, Facultad de Ciencias, Pontificia Universidad Javeriana, Bogotá D.C., Colombia; ^6^Food Chemistry Research Group, High Pressure Laboratory, Departamento de Química. Departamento de Química, Facultad de Ciencias, Universidad Nacional de Colombia, Bogotá D.C., Colombia

## Abstract

Multicellular tumor spheroids are used as models in drug development due to their characteristics simulating in vivo tumors. Likewise, antiproliferative properties of extracts derived from fruits have been widely described. Peels and seeds can be used as a matrix to obtain different compounds. Recently, a study demonstrated the antiproliferative activity from a *P. mollissima* extract (PME) on human colon cancer cells; however, its effect on oral spheroids is unknown. *Objective*. To evaluate the antiproliferative potential of an extract obtained from *P. mollissima* seeds on the spheroid-type-3D culture model of CAL 27. *Methods*. CAL 27-spheroids were treated with three concentrations of PME (10, 50, and 100 *μ*g/ml). After 72 hr incubation, morphology and cellular changes, cytotoxic and proapoptotic effect, gene expression, and metastasis were determined. Additionally, changes in the cell cycle phases responded to the PME concentrations. Comparisons between groups were made through a *U* Mann-Whitney test. *Results.* It was shown that 100 *μ*g/ml PE affects CAL 27 cells proliferation grown in spheroids through cell cycle arrest and gene regulation of p53, HIF 1*α*, and CDH1. However, none of the treatments employed induced MMP9 gene expression. *Conclusion.* Our study shows that PME inhibits the growth and proliferation of oral tumor cells cultured in spheroids through the positive regulation of cell death and metastasis genes.

## 1. Introduction

Oral squamous cell carcinoma (OSCC) is a malignancy that may develop at any anatomical site of the mouth. However, it is more prevalent in the tongue. It is development-related *o* behavioral risk factors like tobacco, alcohol drink, human papillomavirus infection, or genetics. The global incidence of oral cancer was 377,713 new cases in 2020 [1]. Treatment of OSCC is based on surgical resection with or without adjuvant treatment (e.g., radiotherapy or chemoradiotherapy). Despite the advances in diagnostics and new therapeutic approaches, the overall survival rate in most countries ranges between 45% and 50%, and it has not improved significantly over the past few decades [2]. Current treatment has been evaluated initially through monolayer cell culture, but it does not represent the microenvironment tumor [3]. The cell interactions and extracellular matrix components are fundamental in the antitumor therapy response, and these features can be mimicked on a 3D culture model like spheroids.

*Passiflora mollissima* (Kunth), L. H. Bailey, is an exotic fruit native to South America, commonly known as “curuba de Castilla” or “banana passion fruit.” It grows in the Andes region at 1800–3600 meters above sea level in climates with average temperatures ranging from 13 to16°C [4]. The leaves, peel, pericarp, and seeds represent a significant and potential source of bioactive principles, with antihyperglycemic [5], antibacterial [6], antifungal [7], antioxidant [8–10], and chemopreventive properties [11]. Recently, we demonstrated the antiproliferative activity of an enriched seed extract in phenolic-type compounds. In this study, the extract affected HT-29 colon cancer cells (IC50 =  39.29 ± 1.82 *μ*g/ml at 48 h), with minor effects on the viability of normal human colon fibroblast cells growing in monolayer [12].

The present study evaluated the antiproliferative potential of an extract from banana passion fruit seeds on a spheroid-type 3D culture model of CAL 27. The first study revealed an extract enriched in phenolic compounds obtained from *Passiflora mollissima* (Kunth L. H. Bailey) seeds that can reduce cell proliferation and induce changes in the morphology of an oral spheroid. The present study evaluated the antiproliferative potential of extract obtained from banana passion fruit seeds on a spheroid-type 3D culture model of CAL 27.

## 2. Experimental Methods

### 2.1. Chemicals

Ethanol (96%) analytical grade, cyclohexane (suitable for HPLC), and gallic acid were obtained from Sigma-Aldrich Chemical Co. (St. Louis-MO, USA). Folin-Ciocalteu phenol reagent was acquired from Merck (Darmstadt, Germany).

### 2.2. *Passiflora mollissima* Seeds Extract (PME)

Samples of *P. mollissima* fruit were purchased in a local market (http://www.corabastos.com.co) in Bogota D. C., Colombia. The seeds and arils of the fruits were separated from the peels after blanching and pulping processes. The seeds were dried and ground using a knife mill. The PME was obtained by the pressurized-liquid extraction (PLE) method. The process was made in two sequential steps using a commercial ASE 200 device as described in the previous work [10], where the extraction conditions (solvent composition and temperature) were optimized to maximize extraction yield, total phenolic content (TPC), total flavonoid content (TFC), and antioxidant activity. In brief, dried and powdered samples of PME seeds (∼1 g) were mixed with sea sand (∼2 g), and the mixture was loaded into a stainless-steel extraction vessel (11 ml of volume). In the PLE-first step, the sample was defatted using cyclohexane as solvent at 100°C for 20 min. Subsequently, for the PLE-second step, ethanol was used as a solvent at 150°C for 20 min to obtain a polar extract. After the extraction, ethanol was evaporated under a stream of nitrogen at 25°C (TurboVap LV Biotage, Uppsala, Sweden). The extractions were performed in duplicate. The extraction yield and TPC tested the similarity between the extract obtained in the PLE-second step at the present work and the extract obtained in the previous work. The extraction yield was obtained by the mean value from the duplicate experiments considering the ratio between the mass of extract and mass of raw material. The TPC of the extracts was measured by the Folin-Ciocalteu method following the same procedure described in the previous work [10]. A calibration curve was plotted with gallic acid (0–100 g gallic acid/ml EtOH), and the sample TPCs (mean of three replicates) were expressed as gallic acid equivalents (mg GAE/g) on a dry extract weight basis (DB). The similarity was analyzed with a Student's *t*-test for comparisons between two groups, and the differences were considered significant when the *p* value was <0.05.

### 2.3. Cell Lines and Culture Conditions

The cell lines, CAL 27 (ATCC® CRL-2095™) oral squamous cell carcinoma, were cultured in DMEM (Eagle modified by Dulbecco) supplemented with 10% fetal bovine serum (FBS-Gibco) and 1% antibiotics penicillin/streptomycin/amphotericin b (Lonza). The cells were incubated in a humidified atmosphere and 5% CO_2_ at 37°C. The cells were given fresh culture media three times per week and were subcultured at confluence after detaching with 0.25% trypsin-EDTA solution.

### 2.4. Cytotoxicity Screening of PME on Monolayer Cultures

The effect of PME on the viability of the tumor's monolayer culture and normal cells was determined by the Alamar blue assay (BioSource, Camarillo, CA, USA). Cells were seeded in 96-well plates at a density of 5 × 10^4^ and 10 × 10^4^ cells/well, respectively, allowing the attachment for 24 h. A stock solution of extract was prepared in DMSO (100 mg/ml (Sigma- Aldrich-Q3251), and five extract dilutions were prepared in medium (6.25, 12.5, 25, 50, and 100 *μ*g/ml) and were added for 48 h and 72 h. At the end of the treatment period, the culture medium was replaced by 100 *μ*L of resazurin solution (44 *μ*M), the plates were incubated for 4 h, and the fluorescence of resorufin was measured in a microtiter plate reader (530–590 nm, Tecan, Infinite® 200 PRO, Switzerland). Chemotherapeutic drug cisplatin (Alpharma) 10 µM was used as a positive control. The cell viability was expressed as the percentage of live cells relative to untreated control. Dose-response curves of the percentage of cell viability plotted against concentrations of the extracts were constructed. Each experiment was performed as three independent tests, with a coefficient of variation of less than 20%.

### 2.5. Preparation and Generation of Spheroid Cultures

Microplates coated with Ultra-Low Attachment (ULA) surface for 3D cell culture (Corning® Costar®) were used for oral spheroid generation. CAL 27 oral tumor cells were seeded at a density of 2.000 cells/well in ULA 96-well plates in DMEM (Eagle modified by Dulbecco) supplemented with 10% fetal bovine serum (FBS-Gibco) and 1% penicillin/streptomycin/amphotericin b (Lonza). The spheroids were obtained by centrifugation at 1300 rpm (500 g) and culture in a humidified atmosphere with 5% CO_2_ and 37°C. The spheroid formation was evaluated under an inverted microscope on the fourth day after seeding.

### 2.6. Morphological Characterization of CAL 27 Spheroids with Image Analysis

Morphological analysis of the CAL 27 spheroids was carried out with photographs at 20X objective using an inverted Zeiss microscope (Zeiss Imager M2BX) equipped with a monochromatic AxioCam HRm CCD camera. The open-source AnaSP2 [13] and ReViSP30 [14] software tools were used to achieve morphological 2D (diameter, perimeter, and area) and 3D (volume, sphericity, solidity, and convexity) parameters to assess the morphological homogeneity of CAL 27 spheroids. Changes in morphology parameters were measured before and after spheroid PME treatment.

### 2.7. Effect of PME on the Viability of CAL 27 Spheroids

CAL 27 spheroids were treated with three concentrations of PME (10, 50, and 100 *μ*g/ml). Cell viability was evaluated posttreatment (72 h) through Alamar blue assay. Cells without extract with DMSO were considered the survival control. Cisplatin (Alpharma) 10 *μ*M was used as a positive control. After the treatment period, 100 *μ*l medium was replaced with 100 *μ*l resazurin solution at 80 *μ*M and incubated overnight in a humidified atmosphere and 5% CO2 at 37°C. Fluorescence was measured with a microtiter plate reader (530–590 nm, Tecan, Infinite® 200 PRO, Switzerland). Each experiment was performed as three independent tests with 119 biological replicates.

### 2.8. Characterization of Spheroid Morphology with Hematoxylin-Eosin (H&E) Stain

A total of eight CAL 27 spheroids per treatment group were embedded using thromboplastin-plasma cell block technique and fixed in 4% paraformaldehyde (PFA) in phosphate-buffered saline (PBS) 0.1 M pH 7.2 solution. The samples were processed for paraffin blocks, cut into sections of 4–5 *μ*m, and stained with hematoxylin-eosin (H&E) stain. Histological images were captured at magnification 20x with an optical Zeiss microscope (Zeiss Axio Imager A2 GmbH).

### 2.9. Double Staining Apoptosis Assay

Evaluate CAL 27 spheroid cell death; Hoechst 33342/PI double staining (eBiosciences) was used for fluorescence microscopy analysis. This method detects fundamental morphological changes in apoptotic cells related to the compacted state of chromatin and alterations in the plasma membrane permeability. The spheroids treated by 72 h with 10, 50 y, 100 *μ*g/ml of PE, cisplatin, and DMSO were stained with 10 *μ*l Hoechst 33342 and incubated for 15 minutes at room temperature. Then, 5 *μ*l of propidium iodide (PI) was added for 5 minutes. The spheroid staining was kept at room temperature for 10 minutes. The fluorescence intensity was measured using ImageJ software.

### 2.10. Clonogenic Assay

The clonogenic assay was used to determine the ability of single cells to replicate and form colonies following exposure to PME extract and cisplatin 20 *μ*M. Cell suspensions derived from disaggregated spheroids were diluted in 500 *μ*l of culture media, and cells were plated in 6-well plates. Spheroids were disaggregated by incubation in trypsin-EDTA for 15 min, followed by gentle manual agitation with a micropipette. Disaggregated spheroids were rinsed in PBS and suspended in factor 1 : 1 in new media following trypsinization and seeded on 6-well plates. The CAL 27 cells were incubated for 11 days before fixation with methanol and staining with 1% crystal violet solution. Colonies were counted. The number of colonies formed was determined by count using ImageJ analysis software. The assay was made by triplicate.

### 2.11. Real-Time PCR Analysis

The transcriptional activity of HIF-1-alpha, TP53, CDH-1, MMP9, and GAPDH (house-keeping) genes was evaluated by a quantitative qPCR 2-ΔΔCT method using Pfaffl formula [15]. A total RNA from 12 spheroids per group with the Quick-RNA™ Miniprep Kit (Zymo Research) was extracted. Following the manufacturer's recommendations, reverse transcription and qPCR were made with Luna® Universal One-Step RT-qPCR Kit (New England Biolabs NEB). Primers are listed in Table 1.

### 2.12. Cell Cycle Distribution in CAL 27 Spheroid Culture

DNA content and CAL 27 distribution during the cell cycle's G0/G1, S y G2/M phases were estimated through flow cytometry. The CAL 27 spheroids (*n* = 8) were treated (10, 50, 100 *μ*g/ml) for 72 hours with PME, washed with PBS solution, and stained with 5 *μ*L Propidium Iodide for 30 minutes. The relative cellular DNA content was determined with BD Accuri C6 in the FLA-3 channel. Data were analyzed using ModFit LT™ software.

### 2.13. Statistical Analyses

All experiments were done in triplicate and repeated three times. The median and interquartile range of all three experiments are shown. GraphPad Prism software was used to do the graphs. Shapiro-Wilk and Kruskal-Wallis determined the data distribution and statistical significance through SPSS software. Finally, multiple comparisons were done between the treatments and cell type cultures (2D and 3D) for *U* Mann-Whitney test.

## 3. Results and Discussion

### 3.1. Evaluation of PLE-Second Step Extract

The extraction yield and TPC of the PLE-second step extract were 7.11 ± 0.17% and 29.44 ± 0.81 mg GAE/g, respectively. According to previous work [10], the extraction yield and TPC of extract were 6.58 ± 0.14% and 29.99 ± 0.58 mg GAE/g, respectively. Student's *t*-tests showed no significant differences (*p* > 0.005) between the extracts. Recent studies have revealed that the agrifood waste fruits extracts constitute a source of bioactive compounds exploitation with anti-cancer potential [16, 17]. Peels, pulp bagasse, and seeds can be used as a matrix to obtain compounds of interest. The antiproliferative activity of residues from industrial processing (pulping) of the “curuba” fruit, particularly the seeds, has been investigated in colon cancer cells (HT-29) [12] and, as far as we know, for the first time in oral cancer cells (CAL 27) in this study. Interestingly, the pressurized-liquid extract obtained from PME showed a significant cytotoxic effect on oral cancer cells (CAL 27) growing in monolayer at 48 h. It was similarly observed for HT-29 cells, suggesting that it might be a good candidate for the spheroid 3D model evaluation.

### 3.2. PME Extract Decrease Monolayer CAL 27 Viability

**Figure 1:**
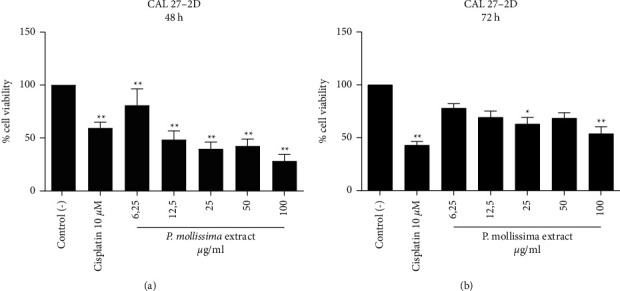
Dose-response effects curve on cell viability of CAL 27 cells treated with PME. The cells were treated with five concentrations of the extract (6.25, 12.5, 25, 50, 100 *μ*g/ml) and 10 *μ*M cisplatin. Results were compared with control (-) (^*∗∗*^*p* < 0.005). Each bar represents the median and interquartile range of 7 independent experiments (*n* = 21) for 48 h and 72 h times.

The high incidence of oral squamous cell carcinoma recurrence and metastasis due to the failure of conventional therapies makes the search of new treatment strategies necessary to enhance the efficacy or reduce the side effects of standard chemotherapy [18]. Despite significant results in cancer research and drug discovery, cancer cells' complex nature and behavior are not reflected in monolayer cultures. In this study, the effect of PME (100, 50, 25, 12.5, and 6.25 *μ*g/ml) and chemotherapeutic drug cisplatin (10 *μ*M) was explored by comparing the viability of CAL 27 cells (Figure 1), and healthy gingival fibroblasts (Supplementary data). It was observed that extract produces cytotoxic effects on the cells in a dose-dependent manner; interestingly, the most effective doses were 50 and 100 *μ*g/ml at 48 and 72 h posttreatment compared to the untreated control cells (Figure 1).

Besides, our results showed that the extract does not produce a cytotoxic effect on gingival fibroblasts, suggesting that it is selective on oral tumor cells. On the other hand, the chemotherapeutic drug cisplatin has a cytotoxic effect on human gingival fibroblast that increases with the treatment time (Supplementary Figure 1). These results confirm the safety of the PME against normal cells and the selectivity against cancer cells compared to the control. Based on this, we chose cytotoxic concentrations 10, 50, and 100 *μ*g/ml in spheroids assays. Thus, cell response to PME and cytotoxic drug cisplatin treatment in 2D cell culture conditions showed a typical dose-dependent response followed by decreased cell viability due to increased extract concentration at 48 h of exposition. Moreover, CAL 27 monolayers growing following extract doses ≥12,5 *μ*g/ml at 72 h showed a partial recuperation of cells from the effect exerted by extract and did not affect the growth of healthy cells. The cytotoxic activity of extract observed on CAL 27 monolayers cells could be due to their phenolic compounds and antioxidant capacity [12].

### 3.3. Characterization of Oral Cell 3D Spheroids

**Figure 2:**
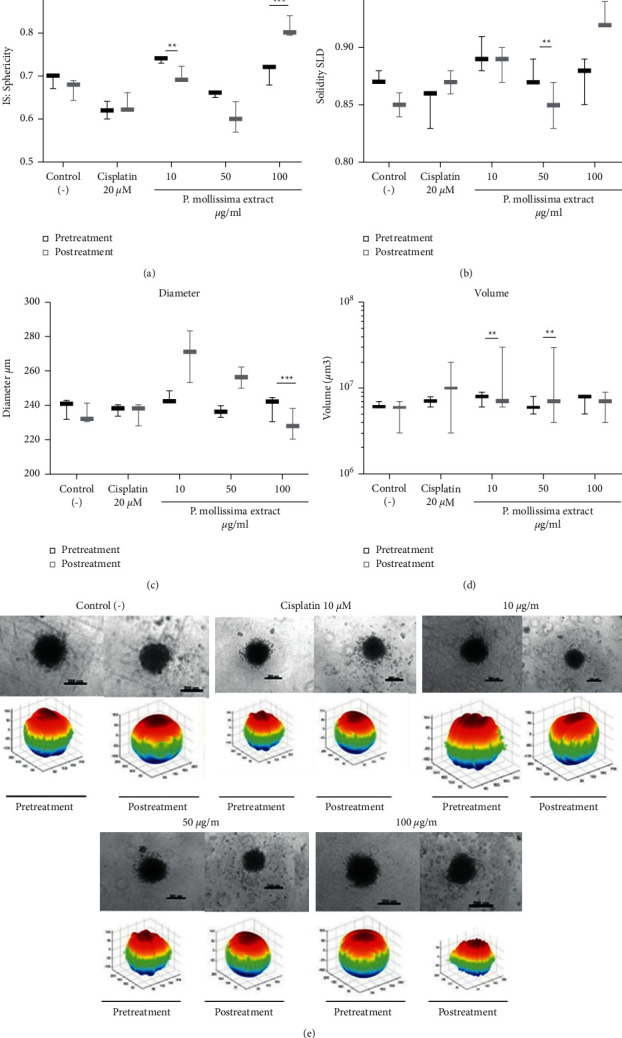
Spheroids of CAL 27 treated and untreated and their three-dimensional reconstruction. Box and Whiskers graphics show the most representative images of the spheroids' morphological changes on parameters measurement before and after the PME treatment (10, 50, and 100 *μ*g/ml) and 20 *μ*M cisplatin 72 h. (a) Sphericity. (b) Solidity. (c) Diameter. (d) Volume. (e) Inverted microscopy images of spheroids and its tridimensional reconstruction with AnaSP software show density color schematically with voxel localization within the three-dimensional image. Scale bar: 200 *μ*m.

Recently, spheroids have been used to evaluate and predict tumor response to chemotherapy/radiotherapy. The screening of active principles with antiproliferative activity is because of their physiological similarities to in vivo solid tumors, and it responds differently to the treatments than the 2D monolayer culture model [19]. To characterize the morphology and size of CAL 27 derived spheroids, we evaluated the influence of cell number (2000-5000-10000 cells) and FBS percentage (2, 5, and 10%) after 72 h of culture (Supplementary Figure 2).  Figure 2 showed that the spheroids with 2000 cells and 10% FBS grow to 219.5∼248.4 *μ*m in diameter and are composed of very densely packed cells with greater sphericity (SI 0.7–1.0), indicating near-perfect circles. We selected this cell density and FBS concentration for a spheroid generation because it produces homogeneous volume and shape to guarantee the homogeneity of our 3D population and treatment with the extract. No differences were observed between the treatment groups with the morphological parameters evaluated (Supplementary Figure 3). Notably, three-dimensional reconstruction showed variations in spheroid shape accompanied by changes in the size and cell distribution (Figure 2). Accordingly, we hypothesized that the 3D size and shape changes reflect the CAL 27 spheroids (Figure 2). We performed Spearman correlation analysis with the morphological measurements obtained for each condition to investigate better these observations. We did not observe strong correlations between morphological parameters and viability results (Data not shown).

### 3.4. PME Induced Morphological Changes on CAL 27 Spheroids

**Figure 3:**
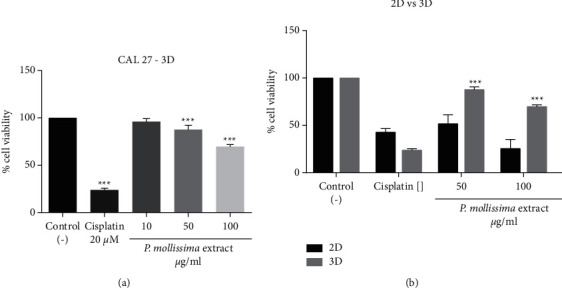
Dose-response curve of effects on cell viability of CAL 27 spheroid treated with PME. The spheroids were treated with three extract concentrations (10, 50, and 100 *μ*g/ml) and 20 *μ*M cisplatin. (a) Viability percent of spheroids treated. Each bar represents the median and interquartile range of 7 independent experiments for 72 h evaluation times. (b) Comparison between viability percentages monolayer and three-dimensional cultures are shown (^*∗∗∗*^*p* < 0.005).

To evaluate the antiproliferative activity of PME on CAL 27 spheroids, we analyzed morphological parameters on cultures treated with 50 and 100 *μ*g/ml of extract and 20 *μ*M of cisplatin 72 h. With brightfield microscopy, quality spheroids appear as translucent balls with a well-defined exterior boundary and a slightly darker core. The data obtained from the Alamar Blue assay showed a significant decrease in cell viability (*p* < 0.005) in spheroids treated with PME and high cytotoxicity with cisplatin (Figure 3(a)).

Cell viability inhibition in monolayer cultures exposed to various concentrations of extract and cisplatin was higher than that in spheroids (*p* < 0.001) (Figure 3(b)), indicating that the CAL 27 spheroids became resistant to treatments. Besides, evident disruption of the spheroid population's architectural structure and a dose-dependent decrease in volume were observed, with PME concentrations (50 and 100 *μ*g/ml) achieving spheroid homogeneous-shaped morphology disintegration. They decreased the clonogenic capacity and growth of the spheroid cells over a 21-day observation period, suggesting that cells response is influenced by the cellular microenvironment or cell-cell contact [20–22]. In agreement with other cancer cell lines, untreated CAL 27 spheroids maintained a compact and spherical structure in the evaluation time [23]. PME and cisplatin treatment-induced changes in spheroids morphology compared to untreated cells related to apparent cell death at 50 *μ*g/ml PME induce the increase in spheroid diameter and volume due to less compact cell packaging and increased cell volume suggesting necrotic cell death; at 100 *μ*g/ml, there are an increase in solidity, decrease in volume, and produced cellular shrinkage, suggesting apoptotic cell death. However, an increased cytotoxicity resistance was observed in 3D cultured cells compared to monolayer cultures indicating that cells in an aggregate structure are much more resistant than cells growing as a monolayer. The treatment resistance in 3D culture can be attributed to limited diffusion through the spheroid and hypoxia, which has been shown to activate genes involved in cell survival [24]. These observations could also be explained by the differences in the expression of heat shock proteins in monolayer and spheroid cultures [25].

### 3.5. Morphological and Cell Changes in Spheroids-CAL 27 Exposed to PME

**Figure 4:**
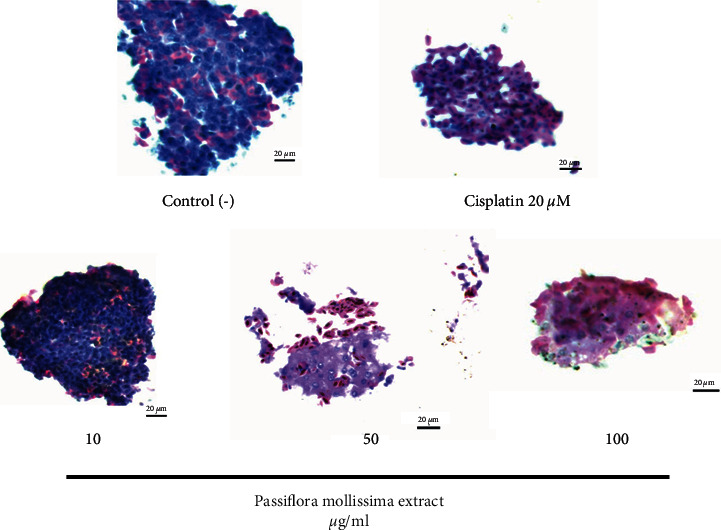
Histological changes of spheroid with H&E staining. It was shown the effect of PME (10, 50, and 100 *μ*g/ml) and 20 *μ*M of cisplatin drug at 72 hours on spheroid morphology. Viable cells with high proliferation and high DNA concentration are dark pink, and necrotic cells show light pink. Scale bar: 20 *μ*m.

The histological stain with hematoxylin and eosin (H&E) showed that the untreated spheroids have a very densely packed cellular network (dark pink) with extracellular matrix deposits (pink). Microscopic evaluation showed morphology integrity at 10 *μ*g/ml of PME with dark pink and violet colorations indicating cells in higher proliferation. On the other hand, spheroids treated with 50 and 100 *μ*g/ml of the extract revealed an evident alteration in spheroid morphology and changes in the H&E coloration. At 50 *μ*g/ml, loss of tissue integrity with increased cell volume and loss of plasma membrane integrity were shown, as well as leakage of cellular content, while at 100 *μ*g/ml, cisplatin was observed, as well as cell shrinkage and matrix compaction. However, both concentrations showed light pink H&E stain, suggesting nonviable cells (Figure 4).

Many cellular morphological characteristics can be related to cell death types. For example, necrosis induces tissue inflammation and is preceded by cell swelling. This increase in cell volume has been mainly due to defective outward pumping of Na + caused by metabolic depletion and increased Na + influx via membrane transporters or nonselective cation channels activated by stress, increasing necrotic cell volume [26]. An apoptotic volume decrease is generally an early event in apoptosis, leading to cell shrinkage due to the outflow of K+ and Cl-from cells [27]. This observation agrees with previous studies that reported a loosening of the spheroid structure and reduction in spheroid volume [28]. However, apoptotic and necrotic cells within PME-treated spheroid remain metabolically active, reducing Resazurin. This phenomenon has been attributed to increased cell volume and mitochondrial mass relative to cell number. Other studies have also demonstrated increased mitochondrial activity during cytotoxic drug etoposide treatment and have linked this with apoptosis [29] and autophagy [30]. Thus, while some cells in the CAL27 PME-treated spheroids could increase in volume, others may shrink due to apoptosis, and yet another group would detach from the spheroid.

### 3.6. PME Induced Cell Death on CAL 27-Spheroids

**Figure 5:**
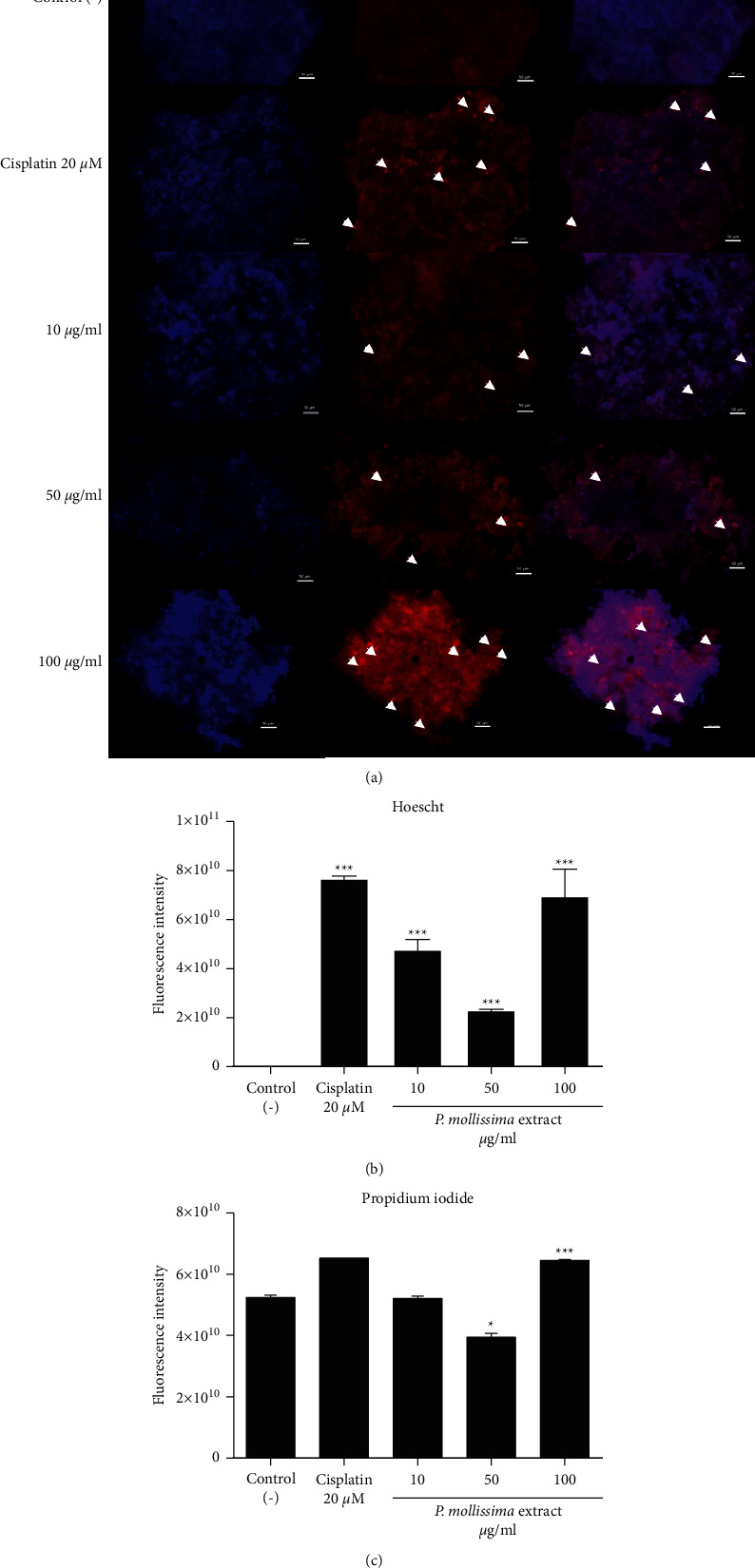
Assessment of 3D spheroid viability changes under PME treatment. Representative images of the proapoptotic effect of PME (10, 50, and 100 *μ*g/ml) and 20 *μ*M cisplatin at 72 h. Spheroids stained with Hoechst and propidium iodide (PI). (A1) Merged image (red and blue dyes). Spheroids control does not show double marking. White arrows (⟶) represent apoptotic cells. [2, 3] Fluorescence intensities of Hoechst and propidium iodide in CAL 27 spheroid treated with different concentrations of PME and 20 *μ*M cisplatin at 72 hours using the ImageJ analysis software. Data are shown as median and interquartile range of 3 independent experiments. ^*∗∗∗*^*p* < 0.05 vs. control.

The mechanism of cell death induced by exposure to the PME was studied by staining with dual fluorescence with Hoechst and Propidium iodide (PI). Hoechst can readily enter living cells and bind to nuclei, whereas PI is membrane impermeant and excluded viable cells. As seen in Figure 5, a significant enhancement in PI fluorescence was observed in spheroids treated with 100 *μ*g/ml of extract and 20 *μ*M of cisplatin, indicating membrane damage in cells. In addition, chromatin compaction, apoptotic bodies, and pyknotic nuclei were observed, which are correlated with a decrease in spheroid size. Apoptotic cells showed typical cell contraction, nuclear condensation, and chromatin cleavage. Conversely, viable cells showed homogeneous chromatin distribution in the nucleus. The spheroids treated with 10 *μ*g/ml showed similarity with the untreated control except for the evidence of few cells in apoptosis (Figure 5).

**Figure 6:**
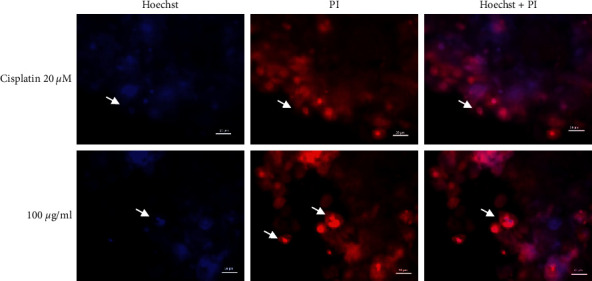
Hoechst and PI double staining for CAL27 spheroids (ROI). A close-up of the most representative images of the proapoptotic effect of 100 *μ*g/ml of PME and 20 *μ*M cisplatin at 72 hours is shown. Some apoptotic bodies (arrows ⟶ white) are observed in the treated spheroid proliferative zone.

To further strengthen the data obtained with fluorescence microscopy, we quantify the fluorescence intensity of Hoechst/PI with ImageJ software. The increase in PI fluorescence intensity was directly proportional to the morphological changes and loss of membrane integrity in cisplatin and extract concentrations. Compared to the untreated control, nuclear chromatin condensation was correlated with Hoechst fluorescence intensity. In contrast, the spheroids treated with 50 *μ*g/ml of PME showed a reduction in PI/Hoechst fluorescence intensity, probably due to loss of spheroid cellularity (Figure 6).

The PI staining intensity increase is observed with cisplatin, and 100 *μ*g/ml of PME is correlated with biological and morphological spheroids changes [31, 32]. The changes are related to membrane permeation, and the increase of fluorescence intensity suggests late apoptosis changes [32]. Additionally, at this time point (72 h), cell viability is reduced as a function of PME dose, and cells are arrested in the G1 cell cycle phase. Otherwise, reduction in PI and Hoechst fluorescence intensity at 50 *μ*g/ml could be due to loss of spheroid cells and suggest that PME-induced cell death (apoptosis or necrosis) may depend on concentration. Furthermore, the antiproliferative activity of PME observed on CAL 27 spheroid cells could be explained by the high content of flavonoids, genuine flavanols, and proanthocyanidin [10] which affects cell metabolic activity and cell cycle progression [12]. Studies showed that the antioxidant capacity of flavonoids (Fisetin) decreases cell viability of the head neck cancer cell lines, including CAL 27, producing changes in cell morphology [33], inducing apoptosis and inhibition of the cell cycle [34].

### 3.7. PME Inhibits Proliferation of CAL 27 Derived Cells Spheroids

**Figure 7:**
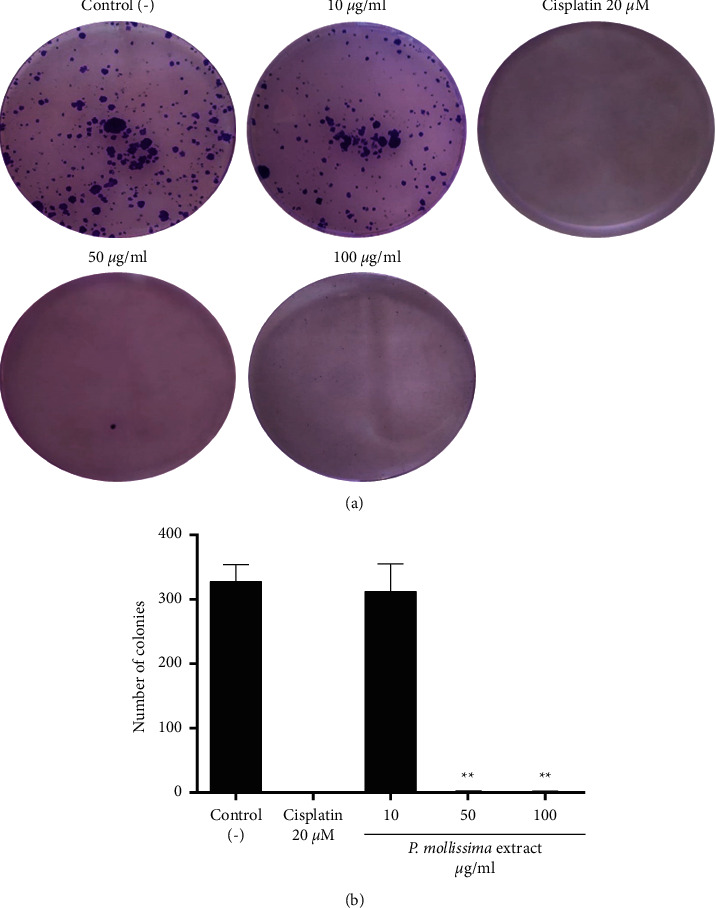
The inhibitory effects of PME and Cisplatin on cell colony formation in CAL 27 spheroids. (a) Representative dishes by colony-forming assay (b) Inhibitory effect of PME (10, 50, and 100 *μ*g/ml) and cisplatin 20 *μ*M on cell proliferation in CAL-27 spheroids and the influence of oral cancer cells on the number of colony-forming cells at 72 hours. The results shown are representative of three independent experiments. ^*∗∗*^*p* < 0.05, control versus PME-treated cells.

We investigate the capacity of spheroid-derived CAL 27 cells to proliferate after treatment with PME (50–100 *μ*g/ml) and 20 *μ*M cisplatin by in vitro clonogenic assay. In Figure 7, untreated spheroids retained their ability to form colonies, while those treated with cisplatin and the highest extract concentrations inhibited colony formation after 21 days of treatment.

### 3.8. PME Induced Modification of Gene Expression and Cell Cycle Arrest

**Figure 8:**
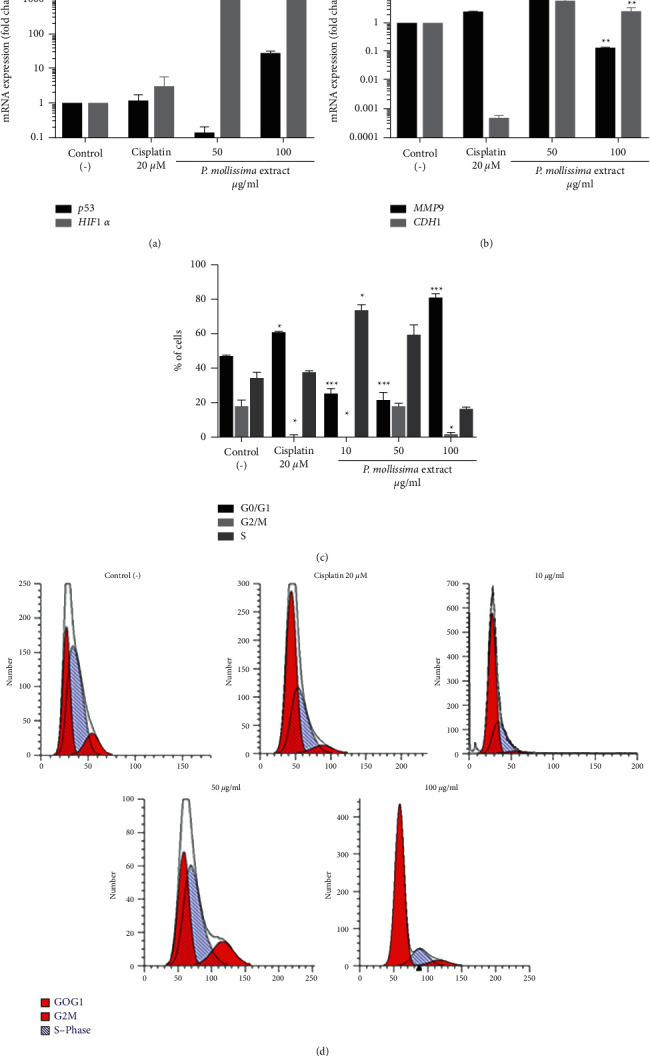
PME induces cell cycle arrest and regulates the expression of genes in CAL-27 spheroids. (a) P53 and HIF1-alpha mRNA expression in CAL 27 spheroids PME-treated (10, 50, and 100 *μ*g/ml) compared to the untreated control and 20 *μ*M cisplatin (b) MMP9 and CDH1 mRNA expression in CAL 27 spheroids. (c) The percentage of cells in G0/G1, G2/M, and S cell cycle phases analysis of CAL 27- spheroids following treatment for 72 h by flow cytometry. (d) Representative histogram of flow cytometry cell cycle. The results shown are representative of three independent experiments. ^*∗∗*^*p* < 0.05 and ^*∗∗∗*^*p* < 0.005, control versus PME-treated cells.

Considering that *P. mollissima* exhibited antiproliferative potential and induced changes in the CAL 27 spheroid morphology and viability, we investigated the expression of genes related to spheroid “wellness.” Our results showed that *P. mollissima* reduced the mRNA expression of p53 at 50 *μ*g/ml and upregulated at 100 *μ*g/ml. In addition, there was a significant increase in mRNA of HIF1-alpha and CDH1 genes with both PME concentrations (50 and 100 *μ*g/ml) (Figure 8).

As indicated in Figure 8, the MMP9 gene is overexpressed at 50 *μ*g/ml and downexpressed at 100 *μ*g/ml compared with the untreated control. Cisplatin drugs showed similar behavior in the expression of p53 and alpha genes and MMP9 genes. In the same way, to understand the mechanism responsible for growth regulation and cytotoxic activity mediated by PME in oral tumor cell spheroids, we analyzed the cell cycle distribution by flow cytometry. PME at 100 *μ*g/ml and cisplatin 20 *μ*M exhibited an increase in G0/G1 population (81.75% and 61.3%, respectively) with a simultaneous decrease in G2/M and S phases, suggesting alterations in cell viability. In contrast, 10 and 50 *μ*g/ml of PME accumulated cells in the S phase. Our finding of HIF-1alpha overexpression on CAL- 27 cells PME-treated reflects the cell-fate decision under these conditions, and at 50 and 100 *μ*g/ml, the expression of HIF-1*α* could induce cell cycle arrest in G1 and G2/M phase, respectively, a classical response of tumor cells to hypoxia [35]. Previous studies have described that PME arrested HT-29 cells in the S and G2/M phases of the cell cycle, which might be mediated by the inactivation of the ubiquitin-like modifier FAT10 signaling pathway involved in cancer survival, proliferation, invasion, and metastasis [12]. Several lines of evidence have indicated that, in transformed cells, hypoxia can provoke apoptosis via the p53 pathway through the inactivation of enzymes responsible for nucleotide synthesis, ultimately inhibiting DNA replication [36, 37]. In this sense, the overexpression of both HIF-1 alpha and p53 at PME 100 *μ*g/ml is consistent with observations suggesting that p53 expression attenuates HIF-1*α* activity, whereas high p53 expression could eliminate HIF-1*α* activity [38].

Interestingly, the overexpression of the E-cadherin gene with PME extract (100 *μ*g/ml) correlates with the findings of p53 expression because E-cadherin is regarded as a tumor suppressor gene that regulates apoptosis signaling via the death receptors DR4 and DR5 [39]. Loss of E-cadherin gene expression has been associated with metastasis in several epithelial cancers [40], and it has been described that E-cadherin acts as a metastasis suppressor by inhibiting the initial dissociation of cells from the primary tumor, reducing the initial steps of tumor cell invasion [41]. Several proteinases have been implicated in E-cadherin cleavage, including MMP-9 [42], and it has been described that MMP-9 expression causes E-cadherin and adherent junction loss in several tumor types [43]. Thus, downexpression of MMP9 with PME extract suggests that extract could favor intercellular junctions in oral cancer cells. In summary, all results confirm the antiproliferative and possible proapoptotic effect of PME at 100 *μ*g/ml on CAL 27 spheroids.

The alterations at the molecular level induced by PME could be explained by its phenolic (29.99 mg gallic acids equivalents/g dry extract weight basis) and flavonoid (0.94 mg quercetin equivalents/g dry extract weight basis) content as well as for its antioxidant activity (6.94 mM Trolox/g of extract and EC50 of 2.66 *μ*g/ml of extract) [10]. Among the previously identified compounds in the extract phenolic acids (i.e., gallic acid, protocatechuic acid, sinapic acid, and syringic acid), phenolic acid derivatives (i.e., vanillic acid glucoside and ecumenic acid), flavonoids (i.e., epigallocatechin, catechin, (epi)fisetinidol, diosmetin, apigenin, and quercetin), and proanthocyanidins (i.e., (epi)gallocatechin-(epi)catechin and (epi)catechin- (epi)catechin) were the most abundant. Recently, several study results have demonstrated those compounds' antiproliferative and proapoptotic properties, particularly flavonoids, in head and neck cancer [44]. And it is well known that natural products are chemical metabolites reservoirs that have a promising future in cancer therapies research. *P. mollissima* seed extract has been evaluated for safety, low cost, and lower toxicity compared to conventional treatment methods. It is added to their high selectivity. A high antitumor capacity of phenols and flavonoids has been described, thanks to their antioxidant and anti-inflammatory capacity [8, 10, 45]. Previous studies using *P. mollissima* in other cells models in monolayer showed that PME promoted a balanced redox from increasing the glutathione levels of the oxidized form, and not in the reduced glutathione form [12].

Thus, our findings provide a basis for further exploration of PME agrifood seed extract as a possible therapeutic strategy for head and neck tumors.

## 4. Conclusions

The present study is the first to demonstrate that *P. mollissima* seed extract can inhibit the growth and proliferation of oral tumor cells cultured in spheroids through cell cycle arrest, positive regulation of p53, HIF 1*α*, and CDH1. Therefore, these wastes constitute a source of bioactive compounds for oral cancer treatment [46].

## Figures and Tables

**Table 1:** Real-time PCR primers.

Gene	Forward	Reverse
GAPDH	GAA GGT GAA GGT CGG GTC	GAAGATGGTGATGGGATTTC
HIF-1*α*	TTAGACTTGGAGATGTTAGC	TTAGCAGTAGGTTCTTGTATT
CDH1	ATC CTG TCT GAT GTG ATG	GTG TTA GTT CTG CTG TGA
MMP9	GTG ACC TAT GAC ATC CTG	CCT CCA GAA CAG AAT ACC
P53	CAG CAT CTT ATC CGA GTG	CAG TGT GAT GAT GGT GAG

## Data Availability

The data of dose curve response of extract effects on healthy fibroblasts, spheroid culture standardization data, and Ct values of the house-keeping genes were used to support the findings of this study and are included within the supplementary information file(s).

## Supplementary Materials

Supplementary Materials
*Figure 1.* Dose-response curve of effects on cell viability of Human Gingival fibroblast treated with PME. *Figure 2.* Spheroids of CAL 27 at different cell number and FBS percentage. *Figure 3.* Measurements spheroids of CAL 27 untreated. *Figure 4.* Variation in Ct values of the house-keeping genes across all the cDNA samples.

## References

[1] Global Cancer Observatory (2021). *Cancer Today*.

[2] Majumdar B., Patil S., Sarode S. C., Sarode G. S., Rao R. S. (2017). Clinico-pathological prognosticators in oral squamous cell carcinoma: an update. *Translational Research in Oral Oncology*.

[3] Fayad W., Rickardson L., Haglund C. (2011). Identification of agents that induce apoptosis of multicellular tumour spheroids: enrichment for mitotic inhibitors with hydrophobic properties. *Chemical Biology & Drug Design*.

[4] Lobo M., Medina C. I. (2009). *Recursos genéticos de pasifloráceas en Colombia. Cultivo, Poscosecha y Comercialización de las Pasifloráceas en Colombia: Maracuyá, Granadilla, Gulupa y Curuba*.

[5] Edwin E., Sheeja E., Dhanabal S. P., Suresh B. (2007). Antihyperglycemic activity of *Passiflora mollissima* bailey. *Indian Journal of Pharmaceutical Sciences*.

[6] Mayta-Tovalino F., Gamboa E., Sánchez R. (2019). Development and formulation of the experimental dentifrice based on *Passiflora mollissima* (Tumbo) with and without fluoride anion: antibacterial activity on seven antimicrobial strains. *International Journal of Dentistry*.

[7] Calderon A., Salas J., Dapello G. (2019). Assessment of antibacterial and antifungal properties and *in vivo* cytotoxicity of Peruvian *Passiflora mollisima*. *The Journal of Contemporary Dental Practice*.

[8] Contreras-Calderón J., Calderón-Jaimes L. S., Guerra-Hernández E., García-Villanova B. (2011). Antioxidant capacity, phenolic content and vitamin C in pulp, peel and seed from 24 exotic fruits from Colombia. *Food Research International*.

[9] García-Ruiz A., Girones-Vilaplana A., León P. (2017). Banana passion fruit (*Passiflora mollissima* (Kunth) LH Bailey): microencapsulation, phytochemical composition and antioxidant capacity. *Molecules*.

[10] Ballesteros-Vivas D., Alvarez-Rivera G., Ibánez E., Parada-Alfonso F., Cifuentes A. (2019). Integrated strategy for the extraction and profiling of bioactive metabolites from *Passiflora mollissima* seeds combining pressurized-liquid extraction and gas/liquid chromatography–high resolution mass spectrometry. *Journal of Chromatography A*.

[11] Chaparro D. C., Maldonado Celis M. E., Urango L. A., RojanoI B. A. (2015). Propiedades quimiopreventivas de *Passiflora mollissima* (Kunth) LH Bailey (curuba larga) contra cáncer colorrectal. *Revista Cubana de Plantas Medicinales*.

[12] Ballesteros-Vivas D., Alvarez-Rivera G., León C. (2020). Foodomics evaluation of the anti-proliferative potential of *Passiflora mollissima* seeds. *Food Research International*.

[13] Piccinini F. (2015). AnaSP: a software suite for automatic image analysis of multicellular spheroids. *Computer Methods and Programs in Biomedicine*.

[14] Zanoni M., Pignatta S., Arienti C., Bonafè M., Tesei A. (2019). Anticancer drug discovery using multicellular tumor spheroid models. *Expert Opinion on Drug Discovery*.

[15] Pfaffl M. W. (2001). A new mathematical model for relative quantification in real-time RT–PCR. *Nucleic Acids Research*.

[16] Ben-Othman S., Jõudu I., Bhat R. (2020). Bioactives from agri-food wastes: present insights and future challenges. *Molecules*.

[17] Osorio L. L. D. R., Flórez-López E., Grande-Tovar C. D. (2021). The potential of selected agri-food loss and waste to contribute to a circular economy: applications in the food, cosmetic and pharmaceutical industries. *Molecules*.

[18] Wang B., Zhang S., Yue K., Wang X. D. (2013). The recurrence and survival of oral squamous cell carcinoma: a report of 275 cases. *Chinese Journal of Cancer*.

[19] Han S. J., Kwon S., Kim K. S. (2021). Challenges of applying multicellular tumor spheroids in preclinical phase. *Cancer Cell International*.

[20] Zanotelli V. R., Leutenegger M., Lun X. K., Georgi F., de Souza N., Bodenmiller B. (2020). A quantitative analysis of the interplay of environment, neighborhood, and cell state in 3D spheroids. *Molecular Systems Biology*.

[21] Franken N. A. P., Rodermond H. M., Stap J., Haveman J., Van Bree C. (2006). Clonogenic assay of cells in vitro. *Nature Protocols*.

[22] Robertson F. M., Ogasawara M. A., Ye Z. (2010). Imaging and analysis of 3D tumor spheroids enriched for a cancer stem cell phenotype. *Journal of Biomolecular Screening*.

[23] Zanoni M., Piccinini F., Arienti C. (2016). 3D tumor spheroid models for in vitro therapeutic screening: a systematic approach to enhance the biological relevance of data obtained. *Scientific Reports*.

[24] Trédan O., Galmarini C. M., Patel K., Tannock I. F. (2007). Drug resistance and the solid tumor microenvironment. *Journal of the National Cancer Institute*.

[25] Song A. S., Najjar A. M., Diller K. R. (2014). Thermally induced apoptosis, necrosis, and heat shock protein expression in 3D culture. *Journal of Biomechanical Engineering*.

[26] Bredel‐Geissler A., Karbach U., Walenta S., Vollrath L., Mueller‐Klieser W. (1992). Proliferation‐ associated oxygen consumption and morphology of tumor cells in monolayer and spheroid culture. *Journal of Cellular Physiology*.

[27] Núñez R., Sancho-Martínez S. M., Novoa J. M. L., López-Hernández F. J. (2010). Apoptotic volume decrease as a geometric determinant for cell dismantling into apoptotic bodies. *Cell Death & Differentiation*.

[28] Ivanov D. P., Parker T. L., Walker D. A. (2014). Multiplexing spheroid volume, resazurin and acid phosphatase viability assays for high-throughput screening of tumour spheroids and stem cell neurospheres. *PLoS One*.

[29] Zamaraeva M. V., Sabirov R. Z., Maeno E., Ando-Akatsuka Y., Bessonova S. V., Okada Y. (2005). Cells die with increased cytosolic ATP during apoptosis: a bioluminescence study with intracellular luciferase. *Cell Death & Differentiation*.

[30] Katayama M., Kawaguchi T., Berger M. S., Pieper R. O. (2007). DNA damaging agent-induced autophagy produces a cytoprotective adenosine triphosphate surge in malignant glioma cells. *Cell Death & Differentiation*.

[31] Crowley L. C., Marfell B. J., Waterhouse N. J. (2016). Analyzing cell death by nuclear staining with Hoechst 33342. *Cold Spring Harbour Protocols*.

[32] Errami Y., Naura A. S., Kim H. (2013). Apoptotic DNA fragmentation may be a cooperative activity between caspase-activated deoxyribonuclease and the poly (ADP- ribose) polymerase-regulated DNAS1L3, an endoplasmic reticulum-localized endonuclease that translocates to the nucleus during apoptosis. *Journal of Biological Chemistry*.

[33] Park B. S., Choi N. E., Lee J. H. (2019). Crosstalk between fisetin-induced apoptosis and autophagy in human oral squamous cell carcinoma. *Journal of Cancer*.

[34] Niedzwiecki A., Roomi M. W., Kalinovsky T., Rath M. (2016). Anticancer efficacy of polyphenols and their combinations. *Nutrients*.

[35] Brown J. M. (2007). Tumor hypoxia in cancer therapy. *Methods in Enzymology*.

[36] Sermeus A., Michiels C. (2011). Reciprocal influence of the p53 and the hypoxic pathways. *Cell Death & Disease*.

[37] Leszczynska K. B., Foskolou I. P., Abraham A. G. (2015). Hypoxia-induced p53 modulates both apoptosis and radiosensitivity via AKT. *Journal of Clinical Investigation*.

[38] Schofield C. J., Ratcliffe P. J. (2004). Oxygen sensing by HIF hydroxylases. *Nature Reviews Molecular Cell Biology*.

[39] Lu M., Marsters S., Ye X., Luis E., Gonzalez L., Ashkenazi A. (2014). E-cadherin couples death receptors to the cytoskeleton to regulate apoptosis. *Molecular Cell*.

[40] Onder T. T., Gupta P. B., Mani S. A., Yang J., Lander E. S., Weinberg R. A. (2008). Loss of E-cadherin promotes metastasis via multiple downstream transcriptional pathways. *Cancer Research*.

[41] Kang Y., Massagué J. (2004). Epithelial-mesenchymal transitions: twist in development and metastasis. *Cell*.

[42] Symowicz J., Adley B. P., Gleason K. J. (2007). Engagement of collagen-binding integrins promotes matrix metalloproteinase-9-dependent E-cadherin ectodomain shedding in ovarian carcinoma cells. *Cancer Research*.

[43] Cowden Dahl K. D., Symowicz J., Ning Y. (2008). Matrix metalloproteinase 9 is a mediator of epidermal growth factor-dependent e-cadherin loss in ovarian carcinoma cells. *Cancer Research*.

[44] Kubina R., Iriti M., Kabała-Dzik A. (2021). Anticancer potential of selected flavonols: fisetin, Kaempferol, and quercetin on head and neck cancers. *Nutrients*.

[45] Viuda-Martos M., Ruiz-Navajas Y., Fernández-López J., Pérez-Alvarez J. A. (2008). Functional properties of honey, propolis, and royal jelly. *Journal of Food Science*.

[46] Klaunig J., Kamendulis L., Hocevar B. (2010). Oxidative stress and oxidative damage in carcinogenesis. *Toxicologic Pathology*.

