# Supplementary material for: Passiflora mollissima Seed Extract Induced Antiproliferative and Cytotoxic Effects on CAL 27 Spheroids
Source: Adv Pharmacol Pharm Sci. 2022 May 31;2022:4602413. doi: 10.1155/2022/4602413 (PMC9174002; doi:10.1155/2022/4602413)
Supplement: Supplementary Materials — Figure 1. Dose-response curve of effects on cell viability of Human Gingival fibroblast treated with PME. Figure 2. Spheroids of CAL 27 at different cell number and FBS percentage. Figure 3. Measurements spheroids of CAL 27 untreated. Figure 4. Variation in Ct values of the house-keeping genes across all the cDNA samples. [file 4602413.f1.pdf]

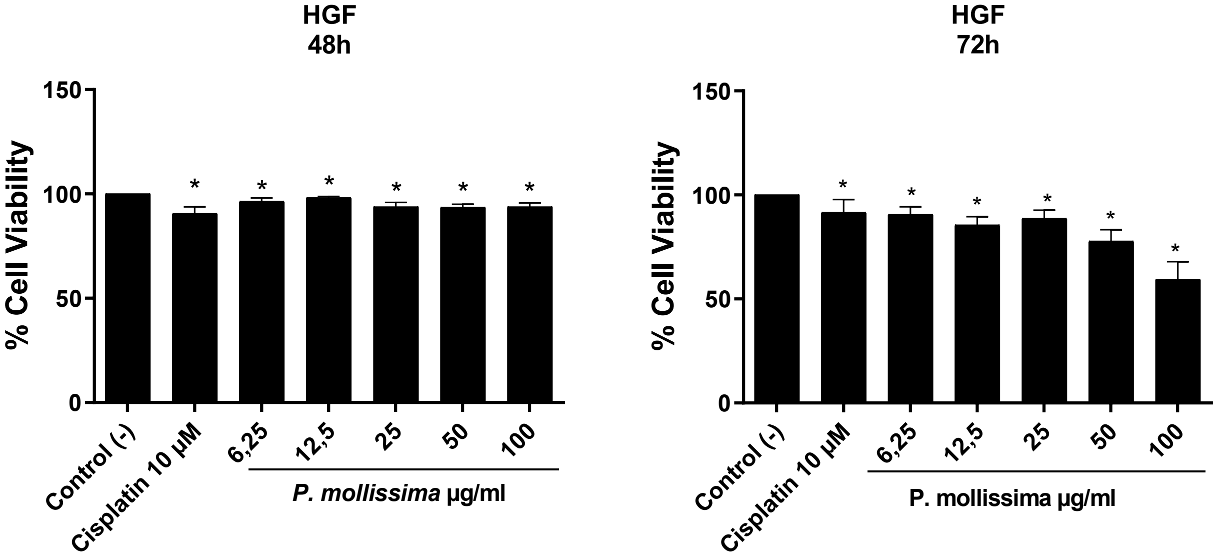

Figure 1- Supplementary. Dose-response curve of effects on cell viability of Human Gingival fibroblast treated with *P. mollissima* extract. The cells were treated with five concentrations of the extract (6.25, 12.5, 25, 50, 100µg/ml) and 10µM cisplatin. Each bar represents the median and interquartile range of 7 independent experiments for both 48h and 72h evaluation times. (***p*<0.005).

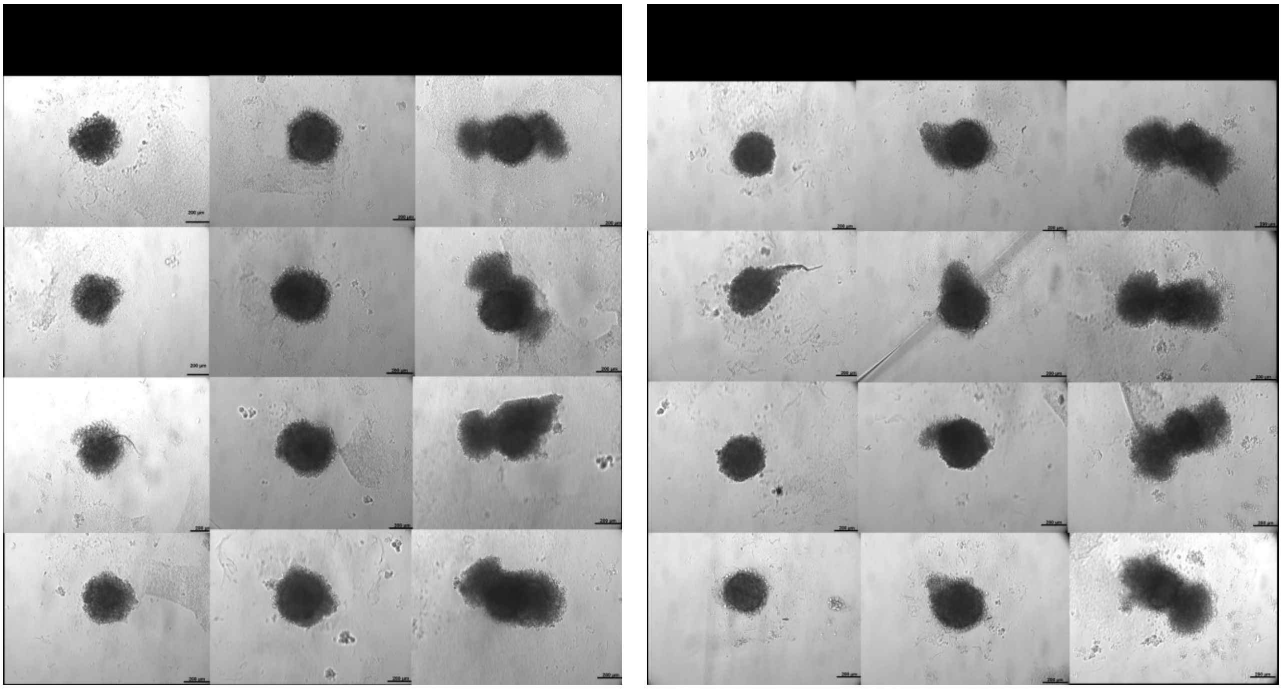

Figure 2. Spheroids of CAL 27 at different cell number and FBS percentage. The most representative images of the spheroids growing at cell number (2000, 5000 and 10000) and FGS percentage (2, and 5%) Scale bar 200µ.

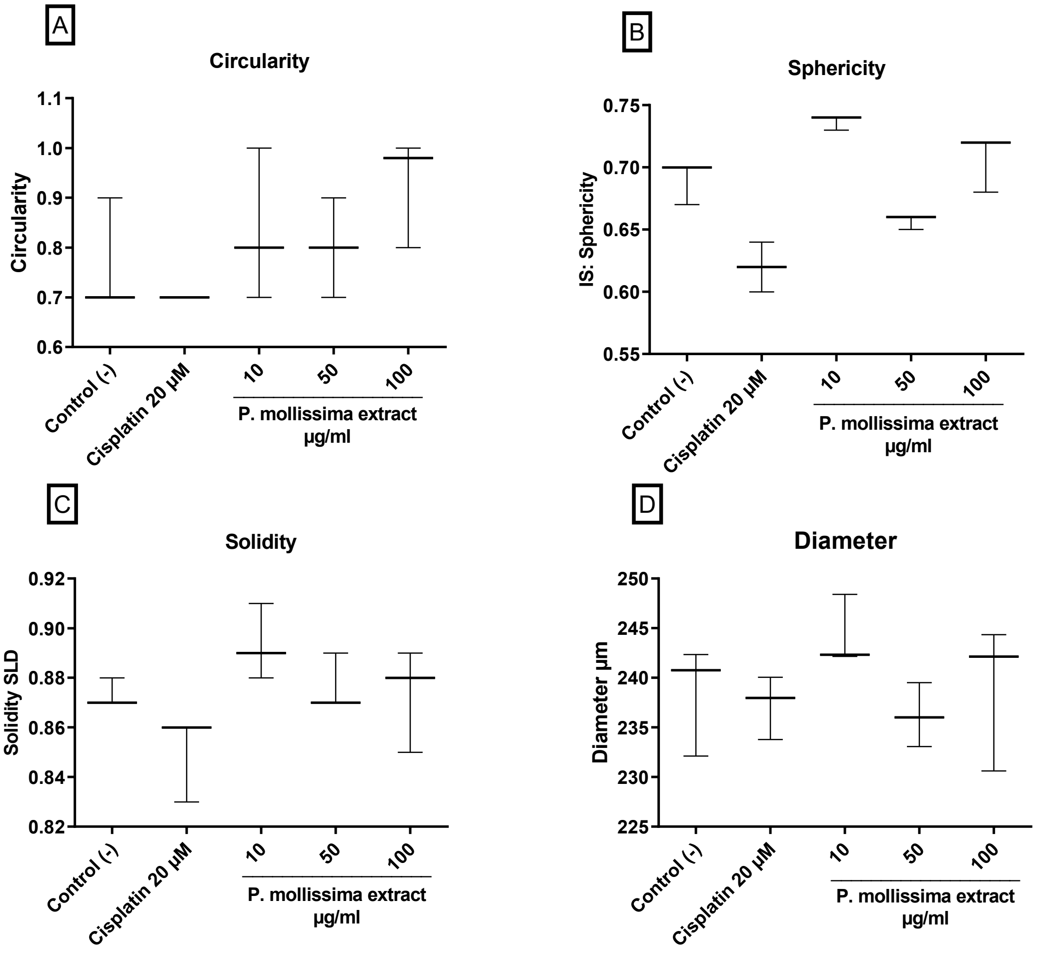

Figure 3. Measurements spheroids of CAL 27 untreated. Graphics show measurement analysis for (A) Circularity, (B) Sphericity, (C) Solidity and (D) Diameter by each treatment group.

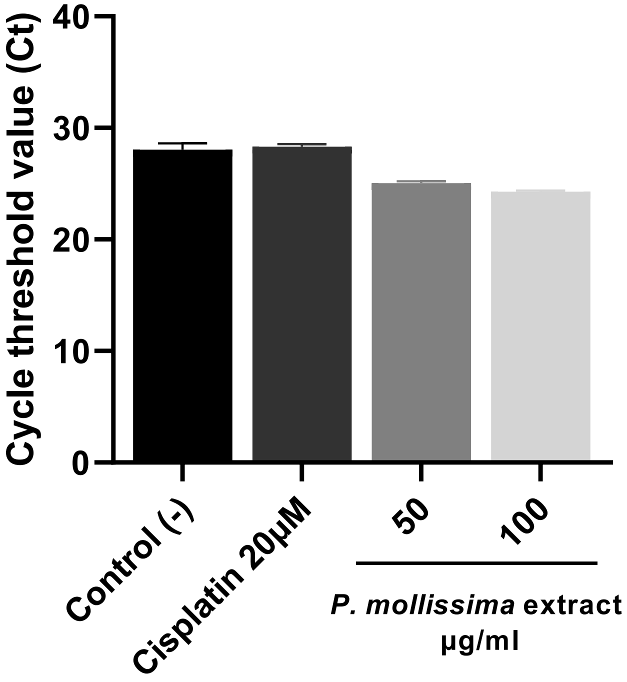

Figure 4. Variation in Ct values of the house-keeping genes across all the cDNA samples. Bar graph showing variation in Ct values for GAPDH house-keeping gene in each treatment.
